# Transplant Trial Watch

**DOI:** 10.3389/ti.2022.10434

**Published:** 2022-04-19

**Authors:** Simon R. Knight

**Affiliations:** ^1^ Centre for Evidence in Transplantation, Nuffield Department of Surgical Sciences, University of Oxford, Oxford, United Kingdom; ^2^ Oxford Transplant Centre, Churchill Hospital, Oxford, United Kingdom

**Keywords:** liver transplantation, hepatocellular carcinoma (HCC), cytomegalovirus, solid organ transplant, randomised controlled trial

To keep the transplantation community informed about recently published level 1 evidence in organ transplantation ESOT and the Centre for Evidence in Transplantation have developed the Transplant Trial Watch. The Transplant Trial Watch is a monthly overview of 10 new randomised controlled trials (RCTs) and systematic reviews. This page of Transplant International offers commentaries on methodological issues and clinical implications on two articles of particular interest from the CET Transplant Trial Watch monthly selection. For all high quality evidence in solid organ transplantation, visit the Transplant Library: www.transplantlibrary.com

RANDOMISED CONTROLLED TRIAL 1 Hepatocellular Carcinoma Progression during Bridging Before liver Transplantation

*by Renner, P., et al. BJS Open 2021; 5(2): zrab005*.

## Aims

This was a substudy of the SiLVER study which examined the link between pretransplant bridging therapy and long-term posttransplant survival.

## Interventions

Participants in the original trial were randomised to receive either a centre specific immunosuppressive regimen (mTOR inhibitor free), or a sirolimus based immunosuppressive regimen.

## Participants

350 liver transplant patients from the SilVER study who underwent one or more hepatocellular carcinoma (HCC) bridging treatments.

## Outcomes

The main outcomes of interest were disease-free survival and overall survival within and outside the Milan criteria.

## Follow-Up

Median follow-up was 5.3 years (inter-quartile range (2.4–6.2 years)).

## CET Conclusion

This manuscript reports a substudy of the SILVER trial (sirolimus in liver transplant candidates with HCC), investigating the relationship between pretransplant bridging therapy and post-transplant survival. The authors report that patients with progression despite bridging therapy had inferior survival, and that those patients with tumours downsized successfully with bridging therapy had inferior outcomes compared to those who had smaller tumours initially. This suggests that downstaging patients with tumours exceeding the Milan criteria with bridging therapy does not improve the probability of survival. Whilst these results are interesting, it is important to remember that the SILVER study was not designed or powered to test the effects of bridging therapy, and bridging therapy used was very variable. Future prospective studies would be needed to further assess the role of response to bridging therapy on post-transplant outcomes.

## Trial Registration

EudraCT: 2005-005362-36; Clinicaltrials.gov: NCT00355862.

## Funding Source

Not reported.

RANDOMISED CONTROLLED TRIAL 2 Maribavir for Refractory Cytomegalovirus Infections With or Without Resistance Post-Transplant: Results from a Phase 3 Randomized Clinical Trial

*by Avery, R. K., et al. Clinical Infectious Diseases 2021 [record in progress].*

## Aims

This study aimed to investigate the safety and efficacy of maribavir compared to investigator-assigned therapy (IAT) for the treatment of with or without resistance cytomegalovirus (R/R CMV) infection in solid-organ transplant (SOT) and hematopoietic-cell transplant (HCT) recipients.

## Interventions

Participants were randomised to either the maribavir group or the IAT group.

## Participants

352 HCT and SOT recipients.

## Outcomes

The primary outcome was confirmed CMV viremia clearance. Secondary outcomes included achievement of CMV clearance and symptom control.

## Follow-Up

16 weeks.

## CET Conclusions

This multicentre RCT investigated the use of Maribavir (a UL97 protein kinase inhibitor) in post-transplant (HCT or SOT) patients with refractory CMV infection. Maribavir was compared to investigator assigned treatment with either valganciclovir/ganciclovir, foscarnet, or cidofovir. CMV clearance was significantly more likely in the Maribavir group (55.7% vs. 23.9%) and demonstrated less nephrotoxicity than foscarnet, and less myelosuppression than valganciclovir/ganciclovir. Whilst unblinded, the study is pragmatic and well designed. There is some variability in included patients (“refractory” patients had to have failed to respond to one first line therapy, but this was not specified in detail) and in the investigator assigned comparator group, but this likely reflects real-world variations in practice. The results encouraging for the use of Maribavir as an alternative, potentially less toxic, alternative to existing therapies in this setting.

## Jadad Score

3.

## Data Analysis

Per protocol analysis.

## Allocation Concealment

Yes.

## Trial Registration

ClinicalTrials.gov—NCT02931539.

## Funding Source

Industry funded.

## Clinical Impact Summary

Treatment of refractory cytomegalovirus (CMV) infection in solid organ transplant recipients is challenging, with existing therapies limited by toxicity and drug resistance. Ganciclovir resistance is frequently seen, and foscarnet is associated with renal dysfunction in around 50% of patients treated (1). Safer, more effective treatments are needed to improve outcomes.

Avery et al. (2) have recently reported the outcomes of a multicentre, phase 3 randomised controlled trial of Maribavir, a novel UL97 protein kinase inhibitor that interferes with CMV DNA replication and encapsidation. The study randomised solid organ or stem cell transplant recipients with refractory CMV infection to Maribavir or investigator assigned treatment (IAT; valganciclovir/ganciclovir, foscarnet or cidofovir). Maribavir-treated patients demonstrated significantly higher clearance of viraemia after 8 weeks of treatment compared to IAT (55.7% vs. 23.9%). This response also appeared more sustained with Maribavir, with more patients achieving viraemia clearance and symptom control through to week 16.

Perhaps as importantly, Maribavir also appeared to have an improved safety profile compared to other agents. Incidence of renal dysfunction was lower than with foscarnet, and neutropenia was less frequent than valganciclovir/ganciclovir. Dysguesia was the most frequently reported side effect in Maribavir-treated patients. Overall, fewer patients discontinued therapy due to side effects than in the IAT group.

The study is pragmatic and well designed. It is not blinded, although this would be challenging given the different routes of administration of the various agents. There is some variability in included patients (“refractory” patients had to have failed to respond to one first line therapy, but this was not specified in detail) and in the investigator assigned comparator group, but this likely reflects real-world variations in practice.

Overall, the results are very encouraging and suggest that Maribavir offers an effective, better tolerated alternative to existing therapies for refractory CMV.
